# Pathways to nurse development and retention: development of an academic/community-engaged partnership

**DOI:** 10.1186/s12912-025-03011-1

**Published:** 2025-04-07

**Authors:** Lakeshia Cousin, Courtney Bowen, Linda Behar-Horenstein, Debra Lyon, Kimberly Martinez

**Affiliations:** 1 College of Nursing, University of Florida, Gainesville, FL USA; 2 Office of Community Outreach and Engagement, UF Health Cancer Center, Gainesville, FL USA; 3 University of Florida Health Shands Hospital, Gainesville, FL USA

**Keywords:** Nursing, Workforce development, Nursing shortage, Health equity, Diversity, Mentorship, Community engagement, Under-represented students (URMs)

## Abstract

**Background:**

A looming nursing shortage is anticipated by 2025 due to 30% of faculty retirement and the global shortage of 13 million nurses by 2030. Addressing this crisis requires innovative strategies that prioritize diversity and address health inequities. This study aimed to develop and assess the pilot implementation of a community-engaged program targeting underrepresented high school students in a southeastern state.

**Methods:**

Evaluation of a nurse development and retention for underrepresented (URM) high school students. Workshop attendance rates were recorded. Program acceptability was measured using the Client Satisfaction Questionnaire-3 (CSQ-3). Changes in participants’ intention to apply to nursing programs were assessed using the General Self-Efficacy Scale (GSE). Registered nurses provided mentoring and offered educational workshops and presentations on nursing career pathways.

**Findings:**

Twenty-one African American students from an underserved high school participated. Attendance rates were high, with 81% attending all workshops. Mean CSQ-3 scores demonstrated high program satisfaction (mean = 11). There was a significant increase in GSE scores from 30.81 to 32.57 (*p* = 0.017), indicating improved self-efficacy to pursue nursing careers.

**Conclusions:**

The study demonstrates that a community-engaged nurse development program was effective as potential approach to address the nursing shortage among URMs and promoting workforce diversity. Mentorship-driven initiatives have the potential to inspire and empower URMs to pursue nursing careers. Further research is necessary to evaluate the program’s long-term impact on workforce development and its scalability to other communities, contributing to the evidence base for community-centered approaches to address the global nursing workforce crisis and advance health equity.

## Introduction

Nursing is one of the largest healthcare professions in the U.S., with over 4 million nurses. Nurses are critical to patient care, often serving as emergency first responders and providers. Consistently ranked as the most trusted profession, nurses are considered the face of the U.S. healthcare system [1]. There is a projected shortage of 78,610 full-time RNs in 2025 and a shortage of 63,720 full-time RNs in 2030 [2]. One of the major barriers to increasing the number of practicing nurses is the availability of highly educated and trained nursing faculty. However, the nursing faculty workforce is also facing a significant challenge. By 2025, it is projected that more than 30% of active nursing faculty—already in place in 2015—will retire [3]. Faculty vacancy rates are currently at an average of 9.75% among schools with budgeted vacancies, and this rate is expected to rise [3]. In fact, 53% of nursing schools report vacant full-time faculty positions [4, 5]. Some estimates suggest that between 10,000 and 20,000 nursing faculty members in the U.S. could retire by 2025.

While the global nursing shortage is well documented, this pilot initiative focuses on the local implementation of a community-engaged mentorship program designed to increase interest in nursing among underrepresented minority (URM) students. For this study, URM is operationally defined as students from racial and ethnic groups historically underrepresented in nursing, including Black/African American, Hispanic/Latino, and Indigenous populations. Specifically, this study examines how structured mentorship and career exploration opportunities can influence URM high school students’ self-efficacy in pursuing a nursing career. Rather than attempting to address the broader nursing workforce crisis, this study provides preliminary insights into how targeted interventions may contribute to diversifying the pipeline of future nurses.

The Community-Engaged Research (CEnR) framework guided the study design and implementation, ensuring collaboration between academic institutions and the local high school. CEnR principles emphasize active community involvement, fostering culturally relevant mentorship, and engaging stakeholders in the co-creation of educational initiatives [6]. Grounded in the CEnR framework, the program incorporates evidence-based interventions, including mentorship by registered nurses, structured workshops to build career readiness, and strategies to foster cultural alignment and professional growth. Community engagement strategies play a critical role in fostering academic and professional success among URM students. Mentorship has been shown to be a significant factor in increasing self-efficacy, academic confidence, and professional development [7–9]. Culturally relevant mentorship and structured career exploration programs are essential for addressing disparities in nursing education and increasing diversity in the profession.

By engaging local high schools in nursing-focused career preparation, this study contributes initial evidence on mentorship-driven workforce initiatives. Throughout the program, CEnR principles were integrated into participant recruitment, program structure, and mentorship activities. For instance, faculty and practicing nurses collaborated with community partners to develop workshops tailored to students’ needs, addressing both academic preparation and social barriers to nursing education. Early exposure to nursing careers, particularly through Certified Nursing Assistant (CNA) training, plays a crucial role in increasing nursing program enrollment and career progression by providing foundational clinical experience and enhancing workforce readiness [10]. This study reinforces the importance of incorporating CNA training into high school curriculum as a strategic approach to building the nursing workforce. Yet, nursing remains a highly sought-after career path, with more qualified applicants than capacity in educational systems. In 2023, U.S. nursing schools turned away 65,766 qualified applicants from baccalaureate and graduate nursing programs due to faculty shortages and limited capacity [11]. While applications for nursing programs continue to be robust, there is limited diversity of qualified student applicants, combined with low retention and graduation rates of URM students in these programs. Accordingly, the National Academy of Sciences Engineering and Medicine’s ‘The Future of Nursing (FON) 2020–2030: Charting a Path to Achieve Health Equity’ report (2021) presents a thorough strategy for enhancing health care quality, while also addressing the systemic barriers that have hindered previous attempts to eliminate health disparities [12]. One of the major aspects of the FON report focuses on creating and supporting an ethos that invites, retains, and graduates URMstudents. While noting that federal responses to these looming retirements may be insufficient, the National Advisory Council on Nurse Education and Practice (NACNEP) has called for innovative solutions to increase and diversify the nursing workforce [1]. One recommendation is to pilot community-engaged research (CEnR) projects aimed at supporting the development of a diverse nurse faculty and preceptor workforce [6]. These innovative approaches could help address the growing demand for qualified nursing faculty, while also fostering a more diverse and resilient nursing workforce to meet the evolving needs of healthcare.

Major healthcare organizations have acknowledged that a culturally diverse nursing workforce is crucial for improving community health. Recruitment of pre-nursing students from the URM community is essential to building a workforce responsive to the needs of diverse populations and better equipped to provide culturally competent care [13–15]. Culturally competent healthcare practitioners are vital to reducing disparities in health outcomes. Actively recruiting underserved high school students can spark interest in nursing as a career and help build a diverse pipeline of nurses, strengthening the workforce for years to come. Mentorship is fundamental, as it empowers students, enhances their self-efficacy, promotes engagement, and provides guidance that shapes their journey toward nursing, strengthening their confidence and commitment to the profession. Mentors often foster an inclusive learning environment where students feel supported and respected and have their needs met. Creating a nurturing, safe place for students to overcome expectations of bias that fosters a sense of belonging can increase URM students’ recruitment and retention in nursing.

Academic partnerships play a foundational role in positioning nurses for practice success and longevity and for recruiting a diverse nursing workforce for the future. Specifically, community-engaged nurse development and retention programs, a variant of community-engaged research (CEnR) represent a promising solution to these challenges [16]. Embedding culturally relevant mentorship opportunities and building career pathways for high school URMs can be accomplished by fostering early exposure to nursing careers. These programs have the potential to mitigate workforce shortages and promote diversity and health equity. CEnR involves collaboration among non-academic and academic partners to address long-term health equity outcomes [17–19]. CEnR is regarded for its emphasis on maximizing the involvement of those affected by the issue under study and for its goal of ameliorating inequitable social conditions [20, 21]. Collaboration with partners from various community organizations, academic institutions, and professionals strengthens recruitment efforts. For example, nearby universities and colleges that have countless mentors and role models, including those from the college’s nursing department and other healthcare-related disciplines, are essential to the recruitment and retention of URM students [22, 23].

This pilot initiative seeks to determine whether this approach is practical and acceptable in a high school setting and whether participants show increased self-efficacy toward nursing careers.

## Methods

### Study design

The study utilized a community-engaged research approach to establish a relationship with a local high school and its school board to examine the facilitators and barriers for students to pursue nursing careers. Collaboration with the school board facilitated entry into the local high school. Discussions with the school board highlighted the absence of a program at the school that directed students toward nursing careers at the local hospital. Following these discussions, the school board sent a letter of support, which provided the necessary approval to implement the nursing pipeline program at the local high school. This approval allowed for direct engagement with students and parents, enabling the research team to conduct workshops and mentorship sessions. A needs assessment was also performed to identify barriers and facilitators influencing students’ ability to pursue nursing careers, providing essential insights for the development of a sustainable pipeline program. Implementation targeted multiple levels: individual, group, institution, and community. At the individual level, the focus was on recruitment, financial assistance, and mentorship to increase the number of nurses in the pipeline. This involved engaging 21 URM high school students from diverse or disadvantaged backgrounds enrolled in CNA/Nursing career workshops to cultivate their interest and skills in nursing. At the group level, programmatic efforts were directed toward admission, progression, and successful CNA training for program participants. The financial feasibility of implementing similar nursing pipeline programs is an important consideration. This program was supported by a $10,000 internal grant, which covered key components, including personnel ($3,000), workshop materials ($2,000), participant incentives ($2,500), travel ($500), administrative costs ($1,000), and community engagement/miscellaneous expenses ($1,000). Providing this cost breakdown can assist institutions in planning for sustainability and replication. Future research should further explore cost−effectiveness and long−term funding strategies to scale similar initiatives.

Workshops were developed and implemented weekly over six weeks based on students’ needs to ensure their successful progression and attainment of CNA certification. The program included six workshops designed to introduce students to nursing careers and address the specific challenges they might face in pursuing a healthcare career. These workshops covered the following topics: (1) an introduction to nursing and its career pathways (2), strategies for academic and personal success in nursing programs (3), financial literacy and scholarship opportunities for nursing education (4), skill−building for patient care, including communication and empathy (5), a day−in−the−life of a nurse featuring mentorship from practicing professionals, and (6) preparing for CNA certification and clinical job readiness. Each session incorporated interactive activities and provided students with tangible resources to guide them toward a career in nursing.

At the institutional level, six faculty and nurses involved in the program underwent comprehensive training to ensure they were equipped to mentor and engage with students effectively. The training program focused on cultural competency, mentorship skills, and the integration of social determinants of health into educational efforts. Nurses also completed workshops on addressing implicit bias, fostering inclusivity, and tailoring mentorship strategies to meet the needs of URMs. These sessions emphasized creating a supportive and empowering environment for students while addressing the specific barriers they might face in accessing nursing education and careers. Lastly, cultural development education was emphasized to address cultural competency and alignment within the institution. Faculty and registered and Advanced Practice nurses from a nearby university hospital affiliated with a Research 1 university served as program mentors.

### Study setting and population

The study was conducted in a northeastern county in Florida. The population consisted of high school students enrolled at a predominantly Black public school. Informed consent was obtained from all participants prior to their involvement in the study. For children under 18 years of age, parental or legal guardian consent was required in addition to the minor’s assent. Although parents and faculty were engaged in the broader implementation of the nursing pipeline program (e.g., mentorship, logistical support), only high school students were included as study participants. The focus of the study was to evaluate the effectiveness of the intervention on students’ self-efficacy and career intentions in nursing. Given this objective, data collection was limited to students to ensure that findings directly reflected their experiences and outcomes. The study received IRB approval from the Institutional Review Board under the protocol number IRB202201598.

#### Sampling and sample size

Sampling involved a purposive selection of high school students from diverse backgrounds who were actively participating in the program. Faculty and parents were not included as study participants because the primary aim was to measure the intervention’s direct impact on students’ career self−efficacy. The sample size was determined based on feasibility considerations within the high school’s medical program, aiming to engage 21 high school students in alignment with the study’s objectives.

### Inclusion criteria

Study participants were exclusively current students who demonstrated an interest in or potential for pursuing a nursing career. Faculty members and parents were not participants but played integral roles in program development and implementation. Efforts were made to include individuals representing diverse perspectives and experiences relevant to the study objectives. The research team included the local public school administration, the community college, a College of Nursing (CON), and a university hospital affiliated with a Research 1 university who worked collaboratively to facilitate program development and implementation. Institutional oversight was provided by the public school administration, while research guidance was offered by CON faculty. The study’s methods were designed to engage with the community, identify barriers and facilitators, and develop a sustainable pipeline for nursing career development. Barriers included a lack of awareness about nursing as a career option, financial constraints preventing students from accessing nursing education, limited exposure to role models in nursing, and the absence of CNA licensing programs at the local high school. On the other hand, facilitators included the presence of a health sciences program at the high school that introduced students to basic healthcare concepts, support from the school board, and the availability of community resources such as mentorship opportunities and financial aid programs.

### Measures

The General Self−Efficacy Scale (GSE) was used to assess self−efficacy among the URM high school students before and after participating in the program. The decision to use the GSE as a measurement tool is grounded in social cognitive theory, which emphasizes self-efficacy as a key determinant of career choices and persistence in academic and professional pathways [24]. In the context of nursing career intentions, self-efficacy is a crucial psychological factor influencing students’ confidence in their ability to succeed in nursing education and navigate career pathways [25]. Developed by Jerusalem and Schwarzer [26], the 10-item GSE measures respondents’ perceived ability to handle difficult tasks and overcome obstacles effectively. Cronbach’s alphas ranged from 0.75 to 0.90, with the majority in the high 80s. The GSE is unidimensional and individuals are asked to rate their level of agreement with each item using a Likert-rating scale where 1 = strongly disagree and 5 = strongly agree [26]. The Client Satisfaction Questionnaire−3 (CSQ−3) was used to evaluate participant satisfaction with the intervention program [27]. The CSQ-3 is a three−item scale designed to measure clients’ satisfaction with services received, focusing on perceived quality, usefulness, and willingness to recommend the program to others. This scale has demonstrated strong reliability and validity across diverse populations, with reported Cronbach’s alphas typically exceeding 0.85. Participants were asked to rate their level of agreement with each item using a Likert scale, where 1 = strongly disagree and 4 = strongly agree, with higher scores indicating greater satisfaction with the program.

### Data analysis

Data were analyzed using paired samples t−tests. Cohen’s d was calculated to quantify the magnitude of change in self−efficacy scores. The significance levels were set at *p* < 0.05. For the CSQ−3, descriptive statistics were used to summarize participant satisfaction scores. Mean scores were calculated to evaluate overall satisfaction with the program.

## Results

Twenty−one African−American students participated in the study. Most were female (77%, *n*=16), and the remainder were male (Table 1). Most (81%, *n* = 17) students attended all six workshops, suggesting high engagement with the program (Table 2). The mean score on the CSQ−3, a measure of program acceptability, was 11 out of 12, suggesting that participants were highly satisfied with the program among participants (Table 3).

**Table 1:** Participant demographics

Characteristic	*N* (%)
Total Participants	21 (100%)
Gender	
Female	16 (76%)
Male	5 (24%)
Race/Ethnicity	
African American	21 (100%)

**Table 2:** Workshop attendance and completion rates

Workshop Attendance	*N*	Percentage (%)
Attended all workshops	17	81%
Did not attend all workshops	4	19%
Total	21	100%

**Table 3:** Client Satisfaction Questionnaire (CSQ-3)

CSQ-3 item	Mean score (SD)	Interpretation
Overall satisfaction with the program	3.33 (0.66)	Higher Scores indicate greater satisfaction
Extent to which program met needs	3.67 (0.58)	Higher Scores indicate needs were met
Willingness to Recommend Program	3.71 (0.46)	Higher Scores indicate a likelihood to recommend
Total Satisfaction Score	10.71 (1.23)	Higher Scores indicate overall program satisfaction

Statistically significant increases in self−efficacy scores were observed from pre−workshop (M = 30.81, SD = 5.25) to post−workshop (M = 32.57, SD = 4.06); t = 2.266, *p* = 0.017. The standardized effect size (Cohen’s 𝑑) was 0.495, with a 95% confidence interval ranging from 0.943 to 0.35 (Table 4). This effect size indicates a moderate improvement in students’ perceived ability to handle difficult tasks and overcome obstacles effectively. These results suggest that participation in the workshops was associated with significant improvements in students’ self−efficacy, highlighting the potential of targeted interventions to enhance educational and career readiness among underrepresented minority high school students in nursing. The post-workshop surveys were administered during the final session of the program, ensuring immediate assessment of participants’ experiences and self-efficacy levels while the program’s impact was fresh in their minds.

**Table 4:** Pre-Post General Self-Efficacy (GSE) scores

Measure	Score/value	Interpretation
Pre-Workshop GSE (Mean ± SD)	30.81 ± 5.25	Self-efficacy before program
Post-Workshop GSE (Mean ± SD)	32.57 ± 4.06	Self-efficacy after program
t-value	2.266	Statistical test result
p-value	0.017	Significance level
Cohen’s d	0.495	Effect size (moderate)
95% Confidence Interval	0.943 to 0.35	Range of effect size

## Discussion

The study findings align with the CEnR framework by demonstrating that collaborative, community−driven mentorship programs can positively influence students’ career confidence. Increased self−efficacy scores among participants reflect the impact of structured guidance and early career exposure in a culturally responsive environment. By fostering early exposure to nursing careers, embedding culturally relevant mentorship, and structured interventions, participants demonstrated significant improvements in self−efficacy and engagement. These findings align with previous studies emphasizing the role of mentorship and early exposure in inspiring underrepresented students to pursue healthcare careers [1, 28]. However, as a single−site pilot initiative with a small sample size, these findings should be interpreted as preliminary rather than indicative of broad workforce transformation [15, 17, 21]. Findings from this study align with existing research indicating that early exposure to healthcare careers during high school is associated with successful completion of nursing programs [29]. Students who develop an early interest in nursing are more likely to persist through the rigorous demands of academic and clinical training, underscoring the importance of interventions that engage students in career exploration at a formative stage [30]. Structured mentoring programs and career pathway initiatives have been shown to enhance students’ self−efficacy, motivation, and preparedness for nursing education, particularly among underrepresented and underserved populations. For instance, mentoring programs targeting adolescents in predominantly Native Hawaiian communities have demonstrated positive effects on student engagement, professionalism, and cultural humility, reinforcing the value of early career exposure [30]. Additionally, interventions designed to promote healthcare career interest among high school students from underserved areas have been identified as a promising approach to addressing workforce shortages and increasing diversity in the nursing pipeline [29].

These findings support the continued development and expansion of nursing pipeline programs, particularly those aimed at students from historically underrepresented backgrounds. Future research should explore longitudinal outcomes to assess the long−term impact of early career exposure on nursing education completion rates and workforce retention. Additionally, integrating culturally tailored mentorship models may further enhance program effectiveness in fostering a sustainable and diverse nursing workforce. By targeting high school students, this program aimed to strengthen early career commitment, which is a key predictor of long−term success in nursing education.

Rather than addressing the global nursing shortage, this study provides insight into the localized impact of mentorship and career exploration for URM students. Future research should focus on expanding the intervention, testing its scalability in different educational contexts, and assessing long−term outcomes, including nursing program enrollment and career progression. Additionally, longitudinal follow−up studies are necessary to track participants’ career trajectories and determine whether initial increases in self−efficacy translate into sustained engagement in the nursing profession.

This study offers a model for enhancing recruitment, mitigating faculty shortages, and fostering a resilient and representative nursing workforce by bridging gaps in mentorship and academic-practice partnerships. This research highlights the transformative potential of community-engaged approaches in addressing global nursing shortages, advancing health equity, and improving patient care outcomes. Mirroring the tenets of CEnR, this program embedded culturally relevant mentorship opportunities, to build career pathways for high school URMs by fostering early exposure to nursing careers [6]. This partnership was evidenced by collaboration between the healthcare organization and the local school board. While instrumental in initiating the program, further investment and alignment are necessary to ensure sustainability and effectiveness. Similar challenges with inter−organizational partnerships in educational pipeline programs have been noted in previous research [31], underscoring the importance of policy support and funding alignment. One challenge identified in this study was the legislative requirement for registered nurses (RNs) to be employed directly by the school board to teach senior−level content, creating barriers to external RN support. This constraint mirrors previous studies that highlight the complexity of integrating external healthcare professionals into educational systems [1, 3]. Addressing this issue through policy revisions or alternative funding mechanisms could enable students to graduate with CNA licensure, further strengthening the pipeline.

Expanding the mentorship program offers another opportunity for growth. Initiating mentorship earlier, such as in the ninth grade when students enter a Medical Skills Program, could provide a stronger foundation for nursing aspirations. This aligns with studies showing that early career exposure increases interest and retention in healthcare fields [32, 33]. Providing continuous mentorship throughout high school and into early CNA careers strengthens trust and solidifies long−term professional relationships, which are crucial for sustained engagement. Additionally, incorporating professional development across all mentorship years would enhance participants’ readiness for nursing careers, offering a comprehensive understanding of the profession.

### Limitations and recommendations

The study was limited to a single high school. Hence, the small sample size (*N* = 21) may limit the generalizability of findings, and results should be interpreted with caution. Additionally, while the GSE scores showed statistically significant increases in post-intervention, the potential influence of confounding variables (e.g., prior exposure to healthcare careers, external mentorship, or academic preparedness) on self-efficacy improvements cannot be ruled out. Future studies should incorporate larger, more diverse samples and control for external factors to better assess the intervention’s impact. Nonetheless, this study highlights the potential of targeted pipeline programs in fostering nursing career aspirations. The study design did not allow for long−term student retention or employment outcomes tracking. These limitations echo findings from similar educational pipeline studies that emphasize the need for longitudinal tracking and robust funding models [23, 32]. Future research should allow for longitudinal assessment of career trajectories, employment rates, and the program’s long−term impact on nursing workforce retention. To build on this program’s success, we propose following recommendations:

1. **Strengthen Partnerships with Local School Boards**
  Establish formal agreements to integrate RNs into teaching roles and shared funding to ensure students graduate with CNA licensure.
2. **Expand Mentorship Scope and Duration**
  Initiate mentorship earlier, perhaps beginning in ninth grade, and extend support through CNA employment, fostering consistent and meaningful professional relationships.
3. **Enhance Program Content**
  Incorporate professional development modules and cultural competency training throughout the mentorship timeline, providing participants with comprehensive preparation for nursing careers. Future iterations of the mentorship program could expand professional development and cultural competency training by incorporating advanced clinical skills workshops, training on healthcare technologies like electronic health records and telehealth, and leadership development modules focusing on conflict resolution and team management.
4. **Advocate for Policy Revisions**
  Propose policy changes to create a full−time equivalent (FTE) RN teaching position dedicated to senior−year CNA content. To mitigate current regulatory barriers and funding gaps, draft a policy brief and engage legislative stakeholders. mitigate To mitigate.

This study demonstrated the critical role of academic−practice partnerships in addressing nursing shortages. It also resulted in a replicable model for implementing a community−engaged nurse development program. Programs such as the one described can enhance workforce diversity, advance health equity, and improve care delivery in underserved communities. The study’s findings concur with Dawson’s [28] report on how high school programs can aid in the recruitment of URM students into nursing.

Scaling such initiatives to include other high school locations could significantly strengthen the nursing profession and mitigate the global nursing shortage, ensuring healthcare systems remain resilient and inclusive. Expanding mentorship, addressing regulatory barriers, and integrating long−term policy changes will further enhance the program’s impact and scalability. By investing in these initiatives, healthcare organizations can develop a resilient and representative nursing workforce to meet the needs of diverse patient populations.

Major healthcare organizations have recommended that a culturally diverse nursing workforce is essential to improving the health of this community [14, 34]. Recruitment of pre-nursing students from the URM population, in particular, is vital to building a diversified workforce sensitive to the community’s needs [28]. Despite increasing diversity in the U.S., the same is not reflected in health care or the nursing workforce. Consequences of limited ethnic diversity in the health workforce may lead to increased risk of patient mistrust, segregation, and persistent racial discrimination for minority groups. Recruiting underserved high school students can foster interest in nursing as a career and foster a pipeline of nurses to enhance the workforce [35, 36]. As described in our study, mentorship is fundamental to this effort, given its potential to shape the student’s trajectory toward nursing as a career [37]. As Dawkins [28] recommended, our study also replicated collaboration with partners from a single site, a school board, a nearby university, and professionals to strengthen recruitment efforts. Recruitment and retention of URM students are important to continuing efforts to follow the Institute of Medicine’s recommendations to increase workforce diversity [13–15].

## Conclusion

This developmental study highlights the potential of a community−engaged nurse development and retention program to foster interest in nursing careers among underrepresented students. By leveraging academic−practice partnerships and embedding culturally relevant mentorship, structured workshops, and professional development opportunities, the program demonstrated high engagement and acceptability among participants. However, given the single−site nature of this study and the small sample size, further research is needed to assess long−term career outcomes and scalability. Challenges such as regulatory barriers and funding gaps must be addressed to ensure the sustainability and scalability of such programs. The integration of the CEnR framework throughout program implementation underscores the value of collaborative, community-focused strategies in nursing workforce development. Future studies should explore how CEnR-based initiatives can be expanded to other educational settings to enhance URM student recruitment and retention in nursing. By investing in initiatives like this, healthcare organizations can explore sustainable mentorship models that enhance career self-efficacy and diversify the future nursing workforce.

## Data Availability

The data supporting this study’s findings are available from the corresponding author, [LC], upon reasonable request.
